# Evaluation of high molecular weight DNA extraction methods for long-read sequencing of Shiga toxin-producing *Escherichia coli*

**DOI:** 10.1371/journal.pone.0270751

**Published:** 2022-07-13

**Authors:** Sandra Jaudou, Mai-Lan Tran, Fabien Vorimore, Patrick Fach, Sabine Delannoy

**Affiliations:** 1 Pathogenic E. coli Unit, Laboratory for Food Safety, Anses, Maisons-Alfort, France; 2 IdentyPath Platform, Laboratory for Food Safety, Anses, Maisons-Alfort, France; University of Texas at San Antonio, UNITED STATES

## Abstract

Next generation sequencing has become essential for pathogen characterization and typing. The most popular second generation sequencing technique produces data of high quality with very low error rates and high depths. One major drawback of this technique is the short reads. Indeed, short-read sequencing data of Shiga toxin-producing *Escherichia coli* (STEC) are difficult to assemble because of the presence of numerous mobile genetic elements (MGEs), which contain repeated elements. The resulting draft assemblies are often highly fragmented, which results in a loss of information, especially concerning MGEs or large structural variations. The use of long-read sequencing can circumvent these problems and produce complete or nearly complete genomes. The ONT MinION, for its small size and minimal investment requirements, is particularly popular. The ultra-long reads generated with the MinION can easily span prophages and repeat regions. In order to take full advantage of this technology it requires High Molecular Weight (HMW) DNA of high quality in high quantity. In this study, we have tested three different extraction methods: bead-based, solid-phase and salting-out, and evaluated their impact on STEC DNA yield, quality and integrity as well as performance in MinION long-read sequencing. Both the bead-based and salting-out methods allowed the recovery of large quantities of HMW STEC DNA suitable for MinION library preparation. The DNA extracted using the salting-out method consistently produced longer reads in the subsequent MinION runs, compared with the bead-based methods. While both methods performed similarly in subsequent STEC genome assembly, DNA extraction based on salting-out appeared to be the overall best method to produce high quantity of pure HMW STEC DNA for MinION sequencing.

## Introduction

*Escherichia coli*, a natural inhabitant of the digestive tracts of humans and animals, is a very diverse species, which comprise commensals as well as various pathogens. Among the latter, Shiga toxin-producing *E*. *coli* (STEC) are responsible for serious gastro-intestinal diseases ranging from aqueous diarrhea to hemorrhagic colitis and hemolytic uremic syndrome.

Besides the Shiga toxin, many virulence factors appear to be involved in STEC pathogenicity. Most of these genes are localized on mobile genetic elements (MGEs) such as bacteriophages and plasmids [1–4]. These genes, which may account for up to 20% of the genome of *E*. *coli* confer a high plasticity to the genome of STEC strains.

Whole Genome Sequencing (WGS) currently represents the best method for high-resolution genome typing, exploration of the ‘mobilome’ and characterization. The number of sequence data in databases has exploded over the last 10 years, however, most genomes deposited are drafts genomes composed of a variable number of fragments or contigs. In particular, STEC genome sequences are particularly fragmented, often around 200 contigs. Short-read sequencing data of STEC is difficult to assemble because of the presence of numerous MGEs and repeated elements in the STEC genome, including ribosomal genes, transposons and insertion sequences. This fragmentation results in a loss of information on MGEs (especially plasmids and phages), horizontal gene transfers, copy number variations, as well as structural variations.

The current development of long-read sequencing is thus of particular interest for STEC genomics. The MinION sequencer can produce very long reads (more than 2 Mbp have been obtained–[5]) which can span the repeated elements and produce closed or almost closed genomes [6].

One drawback of this technology compared to short read sequencing is the requirement for a high quantity of high molecular weight (HMW) DNA [7]. Many DNA extraction methods have been described and evaluated in specific settings [8–10]. It appears however that there is no universal HMW DNA extraction method. The efficiency of the method appears highly dependent on the nature of the sample: carbohydrates composition, nucleotide composition, topology, and association with proteins [11]. Furthermore, choosing the “right” method is a balance of many additional criteria including costs, handling time and user friendliness, which overall evaluation is specific to each situation.

In order to implement third-generation sequencing routinely in our lab, we needed a method that would consistently produce HMW STEC DNA, in sufficient quantity and quality, but which was also fast, practical and suitable for BSL3 work; hence, the use of toxic chemicals requiring specific handling and disposal (*i*.*e*. phenol-chloroform) was prohibited. DNA extraction methods usually used to produce DNA for short-read sequencing often comprise numerous centrifugation steps that can be detrimental to DNA integrity. As the spin-column method is routinely performed in many labs, including our own, it was nonetheless included in this comparison.

In this study, we tested three different methods to extract HMW DNA from STEC for the purpose of MinION sequencing: bead-based, column-based (*i*.*e*. solid phase), and salting-out, and evaluated their impact on STEC DNA quantity, quality and integrity as well as performance in long-read sequencing and subsequent assembly.

## Material and methods

### • STEC strains collection

A total of 83 STEC strains (including 34 strains positive for *eae*, and 49 strains negative for *eae*) from 30 different serogroups and 36 different serotypes were selected from the Anses collection to be sequenced and assembled in this study (S1 Table). Most of the strains selected were of bovine origin isolated in France from dairy products or meat. A few strains were isolated from goat milk and cheese.

### • STEC strains culture

Isolates were stored at −80°C in 20–30% glycerol, revived on TSYe plates (BioMérieux) overnight at 37°C and cultured in 10 mL of BHI medium (BioMérieux) overnight at 37°C and 320 rpm. For each strain, genomic DNA was extracted from 1 mL overnight culture using various DNA extraction and purification kits.

### • Genomic DNA extraction

Three different DNA extraction principles were used: (i) bead-based, using beads from four different suppliers: AMPureXP (Beckman Coulter™) (n = 24), HighPrepPCR (MagBio) (n = 7), NucleoMag™ (Macherey-Nagel™, Fisher Scientific) (n = 6) and MagAttract HMW DNA Kit (Qiagen) (n = 20), (ii) solid-phase using silicium columns (Monarch® Genomic DNA Purification Kit, New England BioLabs®) (n = 10), and (iii) salting-out with isopropanol precipitation (MasterPure DNA extraction and purification kit, Lucigen) (n = 55) (S2 Table). The Monarch and MasterPure DNA extraction kits were used according to the manufacturer’s recommendations, except for the final DNA elution / rehydration, which was performed in 10 mM Tris–HCl (pH 8.5) (EB Buffer, Qiagen), and included an RNAseA treatment (RNAseA 100 mg.mL^-1^, Qiagen) immediately after cell lysis. The procedure for HMW DNA extraction using magnetic beads is described in supplementary material (S1 File). In each procedure, the use of vortex mixer was reduced to a minimum and pipetting steps were performed slowly to limit DNA shearing.

All DNA extracts were kept at +4°C after extraction (storage up to 10 months).

### • Genomic DNA quantification and quality control

To allow appropriate relaxation and refolding, the gDNA was quantified at least 24 hours after extraction with a Qubit 3.0 Fluorometer (Thermo Fisher Scientific) using the Qubit dsDNA BR Assay Kit (Thermo Fisher Scientific), according to the manufacturer’s instruction.

The purity of gDNA was estimated with a NanoDrop ND-2000 spectrophotometer (Thermo Fisher Scientific) by calculating A260/A280 and A260/A230 ratios.

The gDNA integrity was determined with a Tapestation 4200 (Agilent) using Genomic DNA Screentapes (Agilent) following manufacturer’s instructions (S3 Table).

### • ONT MinION sequencing

A total of 66 samples were selected for sequencing, 47 samples extracted with the salting-out method and 19 samples extracted with bead-based methods. Libraries were prepared from 1 to 2 μg input DNA using the SQK-LSK109 Ligation Sequencing kit in conjunction with the EXP-NBD104 and EXP-NBD114 Native Barcode Expansion kits (Oxford Nanopore Technologies, Oxford, UK) in accordance with the manufacturer’s instructions. Libraries (6 to 14 per flow cell) were loaded onto R9.4.1 flow cells (Oxford Nanopore Technologies) and sequenced using the MK1B MinION device (Oxford Nanopore Technologies) for 48 h without live basecalling.

### • Illumina MiSeq sequencing

Libraries were prepared from 1 ng of gDNA using the Nextera XT DNA Library Preparation Kit (Illumina Inc.) following the manufacturer’s instructions. Libraries were sequenced using the MiSeq Reagent Kit v2 (300 or 500-cycles) (Illumina Inc.) on a MiSeq System. The quality of the raw short reads was checked using the CLC Genomic Workbench version 21.0.5.

### • Sequencing data analysis

Basecalling of the raw fast5 data was performed with Guppy basecaller version 4.4.2. Demultiplexing was performed with Guppy barcoder version 4.4.2.

Basic run metrics statistics were calculated using in-house python scripts (S1-S3 Scripts in S2 File) (total number of reads, total number of bases sequenced, average size of reads, median size of reads, maximum read length, minimum read length, read length N50) and Nanoplot version 1.29.0 (mean read quality, median read quality) [12].

Raw MinION reads were assembled using Raven version 1.2.2 [13] with default parameters and Flye version 2.8.1 (—nano-raw; genome size: 5m; iterations: x5) [14]. Hybrid assemblies using MiSeq and MinION reads were assembled using Unicycler version 0.4.8 [15, 16] with default parameters. The contiguity and number of contigs of each genome assembly were assessed using Quast version 5.0.2 [17] without reference genome.

### • Statistical analysis

The PCA analysis was performed on R Studio version 4.0.3 and the following packages: factoextra v.1.0.7 and FactoMineR v.2.4. The ellipse.level parameter was set to 0.95.

All other statistical analyses were performed on R Studio version 1.2.5019.

Descriptive analyses of percentages (DNA yield and DNA purity, DIN) were performed using the average and standard deviation (s.d.). Non-parametric statistical tests were performed with α of 5%. The null hypothesis was rejected when p-values were < 0.05.

The Kruskal-Wallis test was used to analyze yield and purity according to extraction kit or extraction method. Mutual comparison of test groups was performed using a Dunn post-hoc analysis with Holm correction.

The Wilcoxon rank sum test was used to analyze read length and quality metrics as well as assembly contiguity metrics between extraction methods.

The Kendall rank correlation was used to analyze association between read length and STEC assembly contiguity.

### • Data availability

MinION and MiSeq raw sequencing data were deposited in NCBI SRA database under the Bioproject number PRJNA808207.

## Results and discussion

The preparation of high-quality high molecular weight (HMW) genomic DNA (gDNA) is critical to fully exploit the capacity of the MinION sequencing platform. In this study, we evaluated several parameters in order to determine the relative effectiveness of three different STEC genomic DNA extraction methods for MinION sequencing.

A collection of STEC strains (positive or not for *eae*) were selected for DNA extraction (S1 Table). All the strains originated from cattle-related matrix (meat, raw-milk, raw-milk cheese, animal) and had previously been serotyped and characterized by qPCR for the presence of the *stx* and *eae* genes [18, 19].

### • Quantity and quality of the extracted DNA

The yield, concentration, purity, and integrity of the extracted DNA were evaluated (Table 1 and S2 and S3 Tables). The DNA concentrations were determined with the Qubit Fluorometer. We used the NanoDrop absorption spectra to assess sample purity and identify potential contamination such as carbohydrates and extraction chemicals. The A260/280 ratio for pure DNA is expected around 1.8. Contaminants absorbing at 280 nm include proteins and phenol. The A260/230 ratio can indicate possible residual chemical contamination such as EDTA, phenol, guanidine salts, or carbohydrates. An A260/230 ratio close to 2.0 is expected for a DNA preparation free of contamination. Integrity of the gDNA was estimated using the DNA integrity number (DIN) obtained from the Tapestation. A DNA sample with little to no short fragments will have a DIN value of >9. The DIN decreases when degradation of the DNA sample increases. For MinION libraries, DNA samples with DIN values >8 are preferred.

**Table 1:** Performance comparison of DNA extraction methods.

DNA extraction kit	DNA extraction method	Mean DNA yield (ng / ml culture) (sd)	Mean A260/280 (sd)	Mean A260/230 (sd)
AMPureXP	Bead-based	4835 (3044)	1.76 (0.19)	0.49 (0.18)
HighPrepPCR	Bead-based	3280 (1668)	1.61 (0.08)	0.52 (0.07)
NucleoMag	Bead-based	2756 (948)	1.58 (0.05)	0.64 (0.16)
Monarch	Solid phase	3162 (1078)	1.60 (0.43)	1.11 (0.40)
MasterPure	Salting-out	8687 (3462)	2.05 (0.09)	2.00 (0.26)
MagAttract	Bead-based	1582 (764)	2.08 (0.04)	1.47 (0.44)

Yield and purity of the isolated DNA varied between extraction methods (Table 1).

Although it produced HMW DNA the DNA concentration and total yield obtained with the MagAttract kit without optimization were not deemed sufficient for MinION library preparation and it was thus omitted from the subsequent steps and analyses.

**Fig 1:**
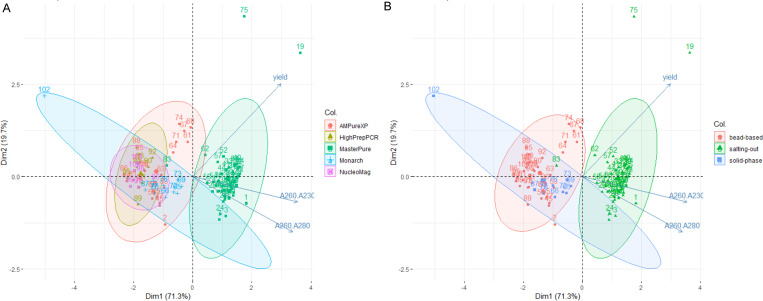
PCA biplot analysis on DNA yield and quality recovered from STEC strains depending on (A) extraction kits and (B) method.

To have a global idea on DNA extraction methods / kits performance we performed a PCA biplot analysis on DNA yields and purity ratios (Fig 1). Two principal components stand out, the first (PC1) representing 71.3% of the variance between data and the second (PC2) which represent 19.7% of the variance. Altogether, these two dimensions represent the three variables DNA yield, and the two purity ratios A260/A280 and A260/A230.

The PCA biplot analysis indicated that the three bead-based extraction kits have similar DNA extraction results (Fig 1A). Consequently, the DNA extractions performed with the beads from the three remaining suppliers were grouped together as “bead-based”. Indeed, the yield, A260/A230 and A260/A280 ratios did not differ significantly between the three suppliers (Kruskal-Wallis test, p-values > 0.05). Except for A260/280 ratio obtained with the AMPureXP beads, which was significantly different from that obtained with the NucleoMag beads (Kruskal-Wallis test, p-value = 0.02768 and Dunn’s post hoc test, p-value = 0.026197) but not from that obtained with the HighPrepPCR beads. It is noteworthy that fewer extractions were performed with the NucleoMag and HighPrepPCR beads compared with AMPureXP beads.

**Fig 2:**
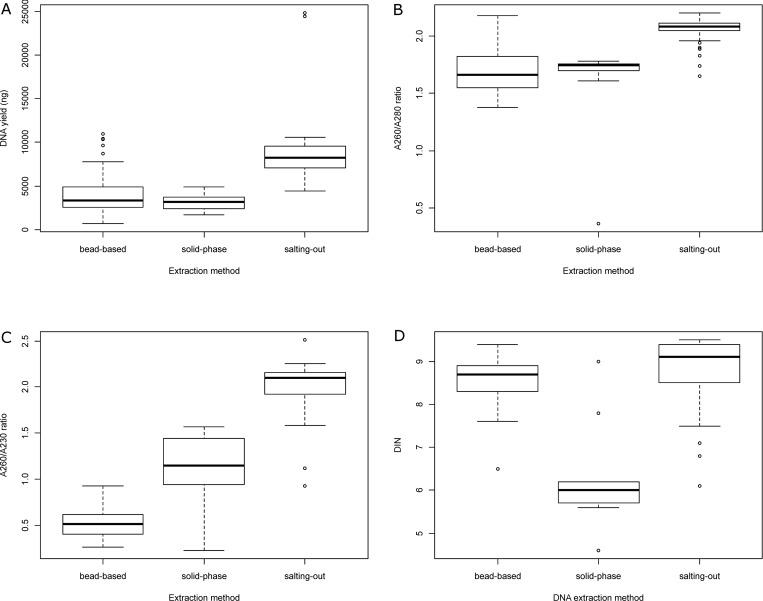
Quantity and quality of the extracted DNA according to the DNA extraction method. (A) DNA yield (ng.mL^-1^ culture) as determined with Qubit dsDNA Broad range assay kit, (B) A260/A280 ratio as determined with Nanodrop, (C) A260/A230 ratio as determined with nanodrop, (D) DIN value as determined with Genomic DNA Screentapes.

The PCA biplot representation showed that the salting-out was the best method to obtain highly concentrated pure DNA compared to the other extraction methods tested in this study (Fig 1B). Taken together the DNA yields were significantly higher with the salting-out method than with the bead-based and solid-phase methods (Kruskal-Wallis test, p-value = 4.025e-11 and Dunn’s post hoc test, p-values 1.6e-09 and 1.7e-06, respectively), which did not differ significantly from each other (p-value = 0.31) (Fig 2A). Similar extraction yields have previously been reported for STEC for these extraction methods [10].

DNA purity as assessed with the A260/280 and the A260/230 ratio differed significantly between the extraction methods (p-values <0.05). No statistically significant differences were observed between the solid-phase and bead-based extraction methods (p-values >0.05). The salting-out method allowed the extraction of significantly purer DNA compared to the solid-phase and the bead-based methods (p-values <0.05). Only DNA extracted using the salting-out extraction method showed A260/280 purity ratio with acceptable values >1.8 and A260/230 purity ratio close to 2.0 (Fig 2B and 2C). The low ratios obtained with the bead-based and solid-phase methods suggest incomplete removal of proteins and organic compounds. The ratios >1.9 obtained with the salting-out method suggest the presence of RNA in the sample, although, based on our experience, the presence of RNA is not detrimental to MinION library preparation.

The DNA extracted with the solid-phase method showed significant degradation of the DNA compared with the other methods (Fig 2D) with a median DIN value of 6.0 (sd 1.3). These results were expected due to the numerous centrifugation steps in this procedure. These DNA were deemed not suitable for MinION sequencing.

Both the bead-based and salting-out methods produced HMW DNA with fragments >60 kb and median DIN values of 8.7 (sd 0.7) and 9.1 (sd 0.8) respectively, indicative of low amounts of genomic DNA degradation (Fig 2D). Integrity of the DNA was checked up to 10 months after extraction and storage at +4°C and showed no sign of degradation with either bead-based or salting-out method.

The bead-based and salting-out methods were the most appropriate methods to yield sufficient DNA amounts and concentrations with appropriate purity and integrity to perform MinION library preparation. To further demonstrate the suitability for long-read sequencing of the genomic DNA obtained, DNA extracted with the bead-based and salting-out methods were subsequently sequenced by the MinION technology.

### • Quality of the sequencing data

Forty-seven samples extracted with the salting-out method and 19 samples extracted with bead-based methods were sequenced with the MinION (S2 and S4 Tables). Quality control checks were performed on the raw data to evaluate the kit’s influence on read quality (mean and median Q score, mean and median read length, read length N50).

Our data indicates that read size distribution is not uniform across extraction methods. The average and median read lengths obtained appeared quite short for some of the isolates, but overall similar read lengths have been reported for STEC and other food strains [20–24]. To avoid short fragments, a more stringent size selection could be applied during library preparation.

**Fig 3:**
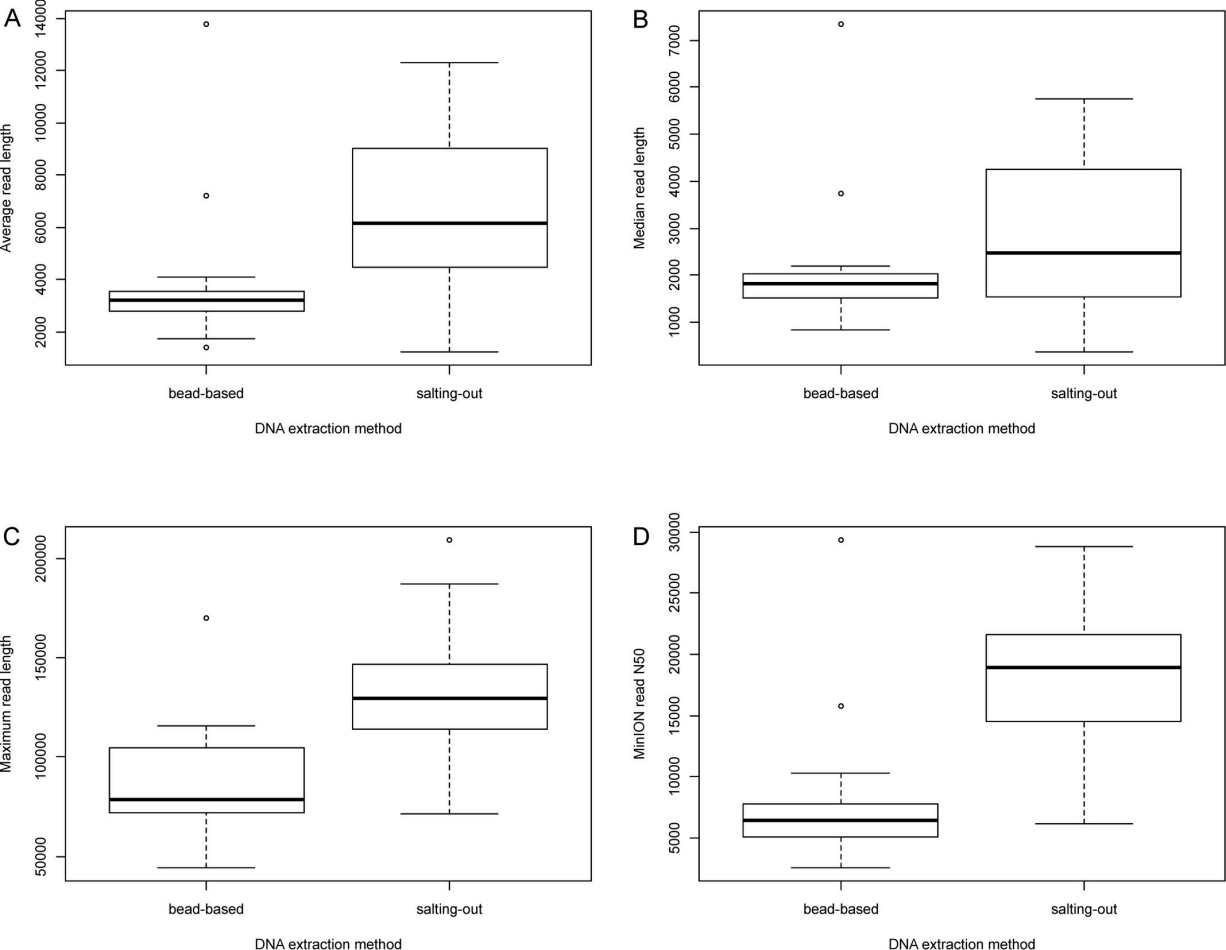
Read length-related metrics of the sequencing data according to the DNA extraction method. (A) Average read length (bp), (B) Median read length (bp), (C) Maximum read length (bp), (D) Read length N50 (bp).

The average read length is significantly higher with the salting-out method (Wilcoxon test, p-value = 9.9e-06), but the median read length is not significantly different (p-value = 0.081) (Fig 3A and 3B).

Similarly, the maximum read length and total read length N50 are significantly higher with the salting-out method (Wilcoxon test, p-values 1.4e-06 and 7.4e-09, respectively) (Fig 3C and 3D). The salting-out method produced the longest fragments (up to 209 kbp) while the bead-based method produced fragments up to 169 kbp.

**Fig 4:**
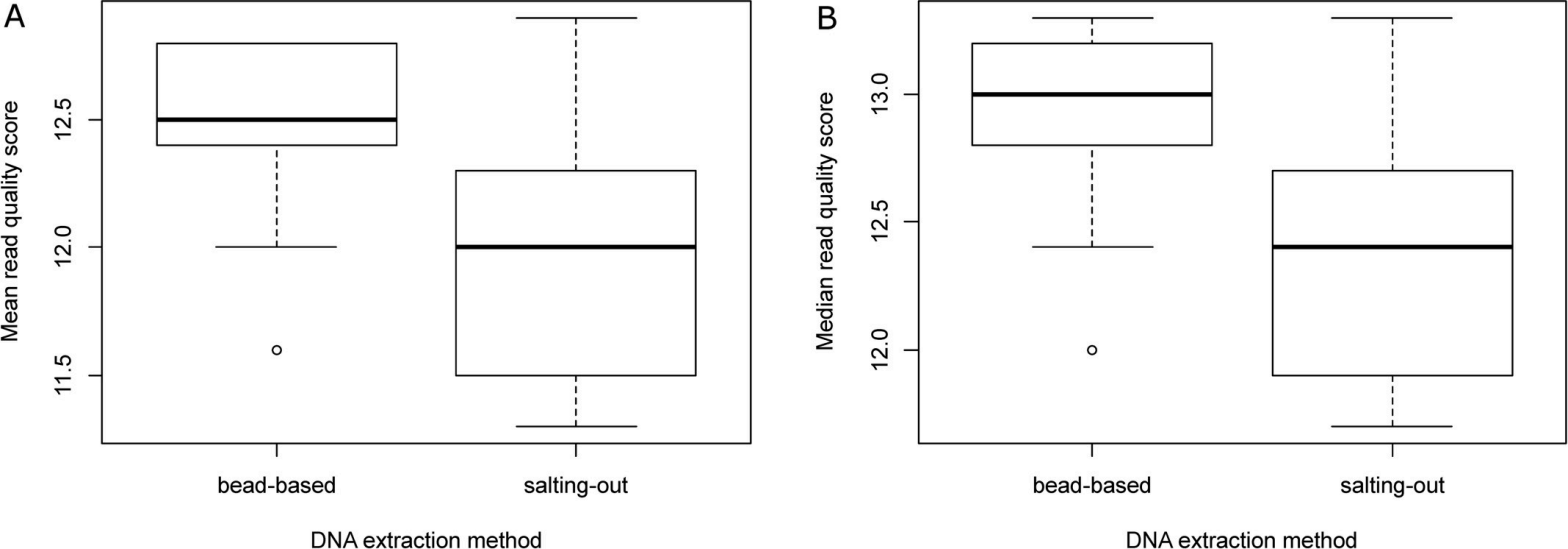
Quality metrics of the sequencing data according to the DNA extraction method. (A) Mean read Q score, (B) Median read Q score.

On the contrary, although the difference appeared modest, both the mean and median Q score were significantly higher with the bead-based method than the salting-out method (Wilcoxon test, p-value = 7.1e-05) (Fig 4), presumably due to the presence of much longer DNA fragments in the salting-out DNA extracts.

### • Contiguity of STEC assemblies

In order to check the influence of the extraction method and more generally of read length on the assembly contiguity we performed crude assemblies of the sequencing data. For each sample (n = 66) we generated three assemblies, one hybrid assembly with Unicycler using MiSeq and MinION reads, and two assemblies with MinION data alone using Raven and Flye. The contiguity of the assemblies were determined with Quast (S4 Table).

When considering the data by extraction method, there was no difference in the number of contigs between the two extraction methods whatever the assembler used (Wilcoxon rank sum tests, p-values > 0.05). While there was no difference between the two extraction methods concerning the N50 of the assemblies generated with Raven and Unicycler (Wilcoxon rank sum tests, p-values > 0.05), the Flye assemblies N50 were significantly longer for the samples extracted with the salting-out method than with the bead-based method (Wilcoxon rank sum tests, p-value = 0.0118).

We studied the correlation between read length, irrespective of the extraction method, and STEC genome assembly contiguity (number of contigs and assembly N50). Our data indicate that within the range of read lengths obtained in this study the influence of read length on assembly contiguity depends on the assembler used.

The quality of the assemblies generated by Raven are not associated with read length within this dataset (Kendall rank correlation tests, p-values > 0.05). On the contrary, the quality of the assemblies generated by Flye and Unicycler appear to be associated with read length to some extent, longer reads generating assemblies with more contiguity (less contigs and a higher N50). Indeed, the average read length is significantly negatively correlated with the number of contigs generated by Flye and Unicycler (Kendall rank correlation test, p-values = 0.04525 and 0.01787 respectively, Kendall’s tau = -0.17178 and -0.20308 respectively), while it is positively correlated with the Flye assemblies N50 (Kendall rank correlation test, p-value = 0.02, tau = 0.19), but not with the Unicycler assemblies N50 (Kendall rank correlation test, p-value = 0.09). Similarly the median read length is significantly negatively correlated with the number of contigs generated by Flye and Unicycler (Kendall rank correlation test, p-values = 0.02263 and 0.00311 respectively, Kendall’s tau = -0.19557 and -0.2535 respectively), while it is positively correlated with the Unicycler assemblies N50 (Kendall rank correlation test, p-value = 0.04946, tau = 0.1655), but not with the Flye assemblies N50 (Kendall rank correlation test, p-value = 0.3729). The total read length N50 is negatively correlated with the number of contigs generated by Flye (Kendall rank correlation test, p-value = 0.04769, tau = -0.16987), but not Unicycler (Kendall rank correlation test, p-value = 0.2203), and it is positively correlated with the Flye assemblies N50 (Kendall rank correlation test, p-value = 0.00024, tau = 0.30909), but not Unicycler (Kendall rank correlation test, p-value = 0.2213).

Overall, samples obtained with both extraction methods performed similarly in the assembly step confirming that both extraction methods are suitable to produce HMW gDNA for MinION sequencing.

## Conclusion

In this study HMW DNA extraction methods were compared based on DNA yield, purity and integrity, as well as MinION read length and quality score and subsequent assembly.

Among all methods tested the salting-out method appears to be one of the best compromise in our hands. It produced highly concentrated HMW STEC DNA of required quality for MinION sequencing. In addition to the high yield, the highest integrity of the DNA obtained from this kit was also evidenced by the longest read length from the sequencing result. It should however be noted that studies [9, 10] have suggested that the salting-out method might be less efficient than other methods for the specific extraction of small plasmids (< 5 kb). This should be taken into account depending on the project. Concerning *E*. *coli*, and STEC in particular, these small plasmids < 5 kb are most likely not involved in virulence or antimicrobial–resistance, but could play a role in bacterial adaptation [25–27].

The bead-based extraction method also produced sequencing-grade HMW STEC DNA and appears a valid alternative to the salting-out method. It is however more tedious to perform with the bead drying time being difficult to standardize.

Finally, the column-based solid-phase method is easy to use and popular but shears DNA, which makes it less suited for long-read sequencing applications. It is also noteworthy that this method generates more waste than the salting-out and bead-based methods.

## Supporting information

S1 Table Characteristics of the STEC strains used in this study. (XLSX)

S2 Table Extraction and sequencing data. (XLSX)

S3 Table DIN values of extracted DNA. (XLSX)

S4 Table Assembly metrics using Unicycler, Flye and Raven assemblers. (XLSX)

S1 File Genomic DNA extraction with Solid Phase Reverse Immobilization (SPRI) beads. (DOCX)

S2 File Python scripts used to calculate MinION reads metrics. (ZIP)
