# Supplementary material for: Evaluation of high molecular weight DNA extraction methods for long-read sequencing of Shiga toxin-producing Escherichia coli
Source: PLoS One. 2022 Jul 13;17(7):e0270751. doi: 10.1371/journal.pone.0270751 (PMC9278759; doi:10.1371/journal.pone.0270751)
Supplement: S1 File — (DOCX) [file pone.0270751.s005.docx]

**Genomic DNA extraction with Solid Phase Reverse Immobilization (SPRI) beads**

Prerequisites

Before starting check that:

- Reagents / consumables needed are present and in sufficient quantity

Materials and reagents :

- SPRI beads (AMPureXP (Beckman Coulter™), HighPrepPCR (MagBio), NucleoMag™ (Macherey-Nagel™, Fisher Scientific)
- DNeasy Blood and tissue kit (Qiagen)
- 70% Ethanol
- Buffer EB (Qiagen) or 10 mM Tris pH 8.5
- RNaseA DNase free (100 mg/ml) (Qiagen)
- Heating block set at 70°C
- Heating block set at 56°C
- Magnetic stand

Remarks:

- All centrifugation steps are carried out at room temperature (15–25°C).
- Buffer ATL and Buffer AL may form precipitates upon storage. If necessary, warm to 56°C for 5 min until the precipitates have fully dissolved.
- **SPRI beads should be at room temperature before use (take out of fridge at least 30 minutes before use)**

Pretreatment for Gram-Negative Bacteria

1. Harvest cells (1 ml of overnight culture) in a microcentrifuge tube by centrifuging for 10 min at 5000 x g (7500 rpm). Discard supernatant.
2. Resuspend pellet in 180 µl Buffer ATL.
3. Incubate at 56°C for 1 h.
4. Add 4 µl RNase A, mix thoroughly
5. Incubate for 10 min at room temperature
6. Add 20 µl proteinase K. Mix thoroughly.
7. Incubate at 56°C for 15 minutes.
8. Add 200 µl Buffer AL to the sample, and incubate at 70°C for 10 min.
9. If sterility has been checked (plate 10 to 50% of tube on TSAYe or PCA plate, incubate overnight), tubes can be taken out of NSB3 lab.
10. Continue with step 1 of “Purification of Total DNA from Bacteria”

Purification of total DNA from bacteria

1. Add 1X to 1.8X of SPRI beads and mix gently by inverting 10 times
2. Incubate for 10 minutes at room temperature
3. Place the plate or tubes on a magnetic stand for 5 minutes.

*NOTE: Use this time to prepare 70% ethanol*

1. Discard supernatant. Avoid disturbing the pellet.
2. Add **1 mL** of 70% Ethanol solution without disrupting the beads.
3. Incubate 30 sec.
4. Discard supernatant. *(Avoid disturbing the pellet)*
5. Add **1 mL** of 70% Ethanol solution without disrupting the beads.
6. Incubate 30 sec.
7. Discard all of the supernatant. *(Avoid disturbing the pellet)*
8. Let air-dry for a maximum of **10 minutes** on the magnetic stand. *(Beads should not be over-dried: should not have a cracked appearance)*
9. On the magnetic stand, “rehydrate” the beads with 60uL of RNAse/DNAse-free water (or EB buffer)
10. Remove the plate from the magnetic stand and mix by pipetting.
11. Incubate at room temperature for 5 minutes.
12. Place the plate on the magnetic stand for 2 minutes.
13. Carefully transfer the supernatant into a new tube or plate. *(Avoid disturbing the pellet.)*
14. Store the samples at **+4°C** for up to a month.
